# Urethral Myiasis Associated With an Indwelling Urethral Catheter in a Male Patient

**DOI:** 10.7759/cureus.100557

**Published:** 2026-01-01

**Authors:** Sotirios Gatsos, Anastasios Christakakis, Olena Kryzhanovska, Ioannis Manolitsis, Christos G Athanassiou

**Affiliations:** 1 Urology, Aberdeen Royal Infirmary, Aberdeen, GBR; 2 Urology, Agios Dimitrios General Hospital, Thessaloniki, GRC; 3 Geriatrics, University Hospitals Dorset, Bournemouth, GBR; 4 Entomology, University of Thessaly, Volos, GRC

**Keywords:** indwelling urinary catheter, myiasis infestation, urethral disease, urinary tract, urinary tract infection

## Abstract

Human myiasis is an infestation caused by fly larvae. Although cutaneous myiasis is the most common form, involvement of the urogenital tract is rare. We present a case of urethral myiasis in an elderly male with a long-term indwelling urethral catheter. The patient presented to the emergency department of a tertiary hospital in Northern Greece with urethral pain. Approximately twenty larvae, each measuring about 10 mm, were removed manually. There were no signs of deeper tissue involvement or bladder infestation. The patient received a short course of oral broad-spectrum antibiotics and reported complete symptom resolution within ten days. No further larvae were identified on repeat evaluation. Retrospective assessment of photographic material by an entomologist suggested that the larvae likely belonged to the Calliphoridae family. To our knowledge, this is the first reported case of urogenital myiasis in Greece. Simple manual removal of the parasites, coupled with a broad-spectrum antibiotic, was a sufficient treatment modality. Timely recognition of parasites and reporting of such cases could help build a better understanding of the natural history of the disease and inform appropriate therapeutic management.

## Introduction

Myiasis is a parasitic disease affecting vertebrates, including humans, caused by larvae of Brachycera (Diptera), which are the immature stages of flies. The exact incidence of human myiasis is unknown, and most scientific data consist of case reports or case series, with up to 37 different fly species reported as causal agents [1]. Geographical distribution includes any area with endemic fly populations, most commonly in tropical or warm temperate climates; however, sporadic cases can occur in nonendemic regions, often involving patients with a recent travel history [2]. Known risk factors include poor hygiene, poverty, living in areas with high fly population densities, older age, mental or physical disability, and the presence of open wounds [1].

Francesconi and Lupi classified myiasis into sanguinivorous, cutaneous, wound, or cavitary types according to the affected tissue, with cavitary myiasis including urogenital involvement [3]. Sanguinivorous myiasis refers to superficial attachment of hematophagous ectoparasitic larvae [4]. Cutaneous myiasis has two subtypes: furuncular, in which larvae penetrate the host’s skin and remain in a subdermal cavity until they mature and emerge, and creeping, in which larvae invade deeper tissues after skin penetration, producing a linear lesion [5]. In wound myiasis, oviposition and infestation occur in preexisting wounds, whereas cavitary myiasis occurs in natural body orifices (e.g., nasopharyngeal, urogenital, intestinal, ocular, or aural) [3,5].

Myiasis can also be classified based on the parasite’s life cycle: obligatory, if the parasite completes its life cycle in the host’s living tissues; facultative, if it can initiate myiasis under certain circumstances but normally feeds on decomposing organic matter; and pseudomyiasis, if the parasite cannot complete its life cycle in the host [3]. Urogenital myiasis is a rare or possibly underreported condition, primarily affecting female patients, with a wide variety of fly species, anatomical locations, and clinical manifestations [6,7].

We report a case of male urethral myiasis associated with an indwelling urethral catheter.

## Case presentation

**Figure 1:**
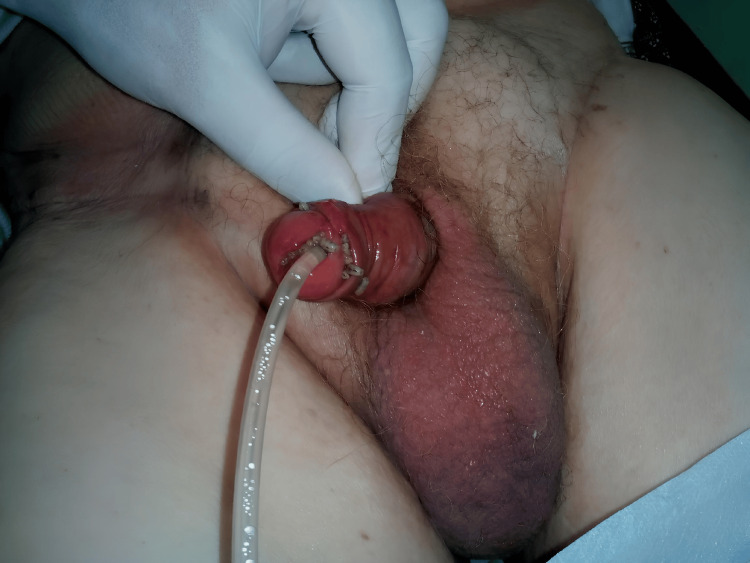
Clinical presentation of urethral myiasis Larvae are visible emerging from the urethral meatus, surrounding the urinary catheter, and accumulating at the coronal sulcus.

An 87-year-old man presented to the emergency department of a tertiary-care urology clinic with worsening urethral pain. Symptoms began approximately 24 hours after a routine indwelling urethral catheter replacement, during the summer months when local fly populations are high. His medical history included well-controlled type 2 diabetes mellitus treated with metformin, with hemoglobin A1c levels of 48 mmol/mol. He had previously undergone an open radical prostatectomy for localized prostate cancer, with no evidence of disease recurrence. Due to urinary retention secondary to an anastomotic urethral stricture, he had required an indwelling urethral catheter for the past two years. Additionally, the patient’s family reported progressive cognitive and motor impairment over the past decade, consistent with possible dementia. On clinical examination, he was afebrile, with stable vital signs and no abdominal or flank tenderness. Inspection of the external genitalia revealed multiple parasitic larvae around the glans and within the urethral meatus (Figure 1).

**Figure 2:**
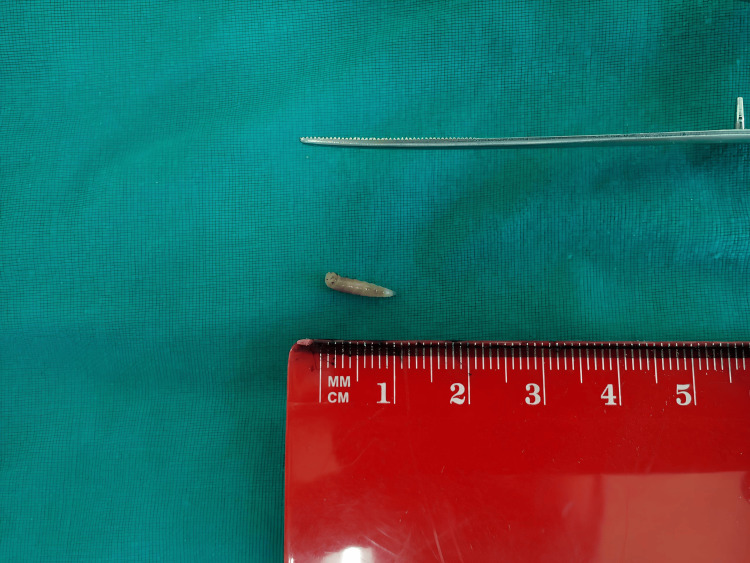
Macroscopic view of an alcohol-preserved larva (~10 mm) Visible features include body segmentation, posterior spiracles (left end), and mouthparts (right end).

Approximately 20 larvae, each about 10 mm in length, were removed manually (Figure 2).

Manual bladder irrigation did not yield additional parasites, and abdominal ultrasonography was unremarkable. The patient was started on an oral broad-spectrum antibiotic (sultamicillin 750 mg twice daily for six days). His catheter was replaced the following day to prevent the potential introduction of residual larvae into the bladder. Blood investigations were within normal limits, with a normal white blood cell count and only a mildly elevated CRP of 12 mg/L. The patient reported immediate symptom improvement, with no further passage of larvae.

Three days later, he re-presented with recurrent mild urethral pain. Clinical examination and contrast-enhanced CT (CT urogram) revealed no evidence of ongoing parasitosis (no visible larvae or glans ulceration and no filling defects in the bladder or upper urinary tract). Given the mild and self-limiting nature of symptoms, cystoscopy and antiparasitic therapy were deferred. At the three-month follow-up, the patient remained asymptomatic with no recurrence.

Parasite identification could not be performed due to failure to preserve a specimen. An expert entomologist retrospectively assessed photographic material, suggesting that the larvae likely belonged to the Calliphoridae family; however, precise identification was not possible.

## Discussion

The present case represents a rather unusual instance of myiasis, as it is one of the few reported cases of urogenital myiasis in a European country, affecting a male patient without a recent travel history. In a systematic review of urogenital myiasis, out of a total of 59 cases in the literature, 19 involved male patients, and 13 were of European origin [7]. The absence of a preserved specimen is a significant limitation, as species identification allows for a more accurate assessment of pathogenicity, invasiveness, and management decisions.

The most probable mechanism of infestation is direct oviposition by a fly at the external urethral orifice, an event that could have occurred during or shortly after catheter exchange. This timeline aligns with the typical hatching period of Calliphoridae eggs, which is approximately 24 hours [8]. The patient’s impaired mobility and cognitive decline likely increased his vulnerability by reducing his ability to repel flies. Larvae of the Calliphoridae family can exhibit various behaviors depending on the genus (i.e., *Calliphora*, *Lucilia*, and *Chrysomya*), ranging from saprophagy to facultative ectoparasitism and obligate parasitism [9].

Our patient presented with no signs of deep tissue damage. In two similar cases of urethral myiasis in male patients with Calliphoridae larvae (i.e., *Chrysomya *sp. and *Lucilia sericata *(Meigen)), necrotic areas were present, even requiring debridement [10,11]. However, in those cases, patients reported a longer duration of symptoms before seeking medical attention (seven and three days, respectively), allowing for further tissue damage by the larvae. A recent case report of a male patient with a spinal cord injury and an indwelling catheter in Ethiopia shares many similarities with our case, as manual removal of the parasites combined with a single dose of ivermectin effectively treated the infestation [12].

In our case, the mild clinical presentation and normal CT urogram did not warrant cystoscopy or administration of antiparasitic drugs. Despite the favorable outcome, this approach cannot be universally recommended, as management should also be guided by the species involved and its pathogenic potential. Antiparasitic regimens with ivermectin, albendazole, and clindamycin have been effective in other forms of myiasis [13]. Moreover, cystoscopic evaluation may be warranted in cases with persistent symptoms or suspicion of deep tissue involvement [11].

Secondary bacterial superinfection is a well-documented complication of myiasis, with several cases in the literature describing progression to sepsis [14,15]. Despite the absence of leukocytosis or significantly elevated CRP, we administered a broad-spectrum antibiotic (sultamicillin) to mitigate the risk of bacterial infection. This treatment was empiric, and no urine culture was obtained at the time of initial assessment. The use of broad-spectrum antibiotics is strongly recommended, as fly larvae are known vectors of multiple microbial species that can cause urinary tract infections via breaches in the urethral mucosa [16].

As previously mentioned, larval identification was not feasible in this case, representing the main limitation. Macroscopically, the larva resembles those of the Calliphoridae family. Given the patient’s limited mobility and urban living environment, the differential diagnosis includes other synanthropic fly species, such as *Musca domestica*; however, distinguishing between species requires microscopy. Physician awareness of this condition is crucial, and specimen preservation and recognition, along with prompt consultation with a specialist entomologist, are essential components of management.

Due to its infrequency, particularly in nonendemic countries, urogenital myiasis presents a challenging clinical problem. In addition to clinical interventions, effective pest control measures in urban and suburban areas are crucial, especially where populations of myiasis-causing species are dense. To prevent oviposition, patients and caregivers should be educated on maintaining hygiene and protecting wounds and natural orifices from flies.

## Conclusions

This report describes the first known case of urogenital myiasis in Greece. While manual removal of larvae and a short course of antibiotics were sufficient in this instance, management decisions should take into account the potential virulence of the causative species. Therefore, species identification, ideally with the assistance of an entomologist, plays a pivotal role in guiding overall management. In this case, species identification was not feasible, which represents the main limitation of the report. Increased awareness and accurate reporting are essential to improve prevention and treatment strategies.

## References

[1] Singh A, Singh Z (2015). Incidence of myiasis among humans—a review. Parasitol Res.

[2] Hohenstein EJ, Buechner SA (2004). Cutaneous myiasis due to Dermatobia hominis. Dermatology.

[3] Francesconi F, Lupi O (2012). Myiasis. Clin Microbiol Rev.

[4] Martín-Vega D, Clark B, García-Del Río M, Merino S, Foronda P, Miquel J, Hall MJ (2025). Comparative larval anatomy of the digestive system of three Calliphoridae (Diptera) species that cause different types of myiasis. Acta Trop.

[5] Robbins K, Khachemoune A (2010). Cutaneous myiasis: a review of the common types of myiasis. Int J Dermatol.

[6] Singh A, Kaur J (2019). Occurrence of human urogenital myiasis due to neglected personal hygiene: a review. Trans R Soc Trop Med Hyg.

[7] Faridnia R, Soosaraei M, Kalani H (2019). Human urogenital myiasis: a systematic review of reported cases from 1975 to 2017. Eur J Obstet Gynecol Reprod Biol.

[8] Wang M, Wang Y, Hu G, Wang Y, Xu W, Wu M, Wang J (2020). Development of Lucilia sericata (Diptera: Calliphoridae) under constant temperatures and its significance for the estimation of time of death. J Med Entomol.

[9] Stevens JR (2003). The evolution of myiasis in blowflies (Calliphoridae). Int J Parasitol.

[10] Abe DK, Rosa RT, Dall'Oglio MF, Fróes MH, Almeida BC, Said LA (2009). Urethral myiasis. Braz J Infect Dis.

[11] Salimi M, Goodarzi D, Karimfar M, Edalat H (2010). Human urogenital myiasis caused by Lucilia sericata (Diptera,Calliphoridae) and Wohlfahrtia magnifica (Diptera,Sarcophagidae) in Markazi Province of Iran. Iran J Arthropod Borne Dis.

[12] Yigzaw KT, Engidaw EA, Asfaw BA, Wondemeneh YA, Moges MT, Getahun GM (2025). Myiasis in a spinal cord injury patient with indwelling catheter: a case report from Gondar, Ethiopia. Int J Surg Case Rep.

[13] Patel BC, Ostwal S, Sanghavi PR, Joshi G, Singh R (2018). Management of malignant wound myiasis with ivermectin, albendazole, and clindamycin (triple therapy) in advanced head-and-neck cancer patients: a prospective observational study. Indian J Palliat Care.

[14] Lysaght TB, Wooster ME, Jenkins PC, Koniaris LG (2018). Myiasis-induced sepsis: a rare case report of Wohlfahrtiimonas chitiniclastica and Ignatzschineria indica bacteremia in the continental United States. Medicine (Baltimore).

[15] Lwanga A, Anis M, Ayoubi M, Sharma J, Khosla P (2018). Two cases of myiasis associated with malignancies in patients living in the continental United States. Cureus.

[16] Förster M, Klimpel S, Mehlhorn H, Sievert K, Messler S, Pfeffer K (2007). Pilot study on synanthropic flies (e.g. Musca, Sarcophaga, Calliphora, Fannia, Lucilia, Stomoxys) as vectors of pathogenic microorganisms. Parasitol Res.

